# Novel Porcine Epidemic Diarrhea Virus Variant with Large Genomic Deletion, South Korea

**DOI:** 10.3201/eid2012.131642

**Published:** 2014-12

**Authors:** Seongjun Park, Sanghyun Kim, Daesub Song, Bongkyun Park

**Affiliations:** Korea Research Institute of Bioscience and Biotechnology, Daejeon, South Korea (S. Park, S. Kim, D. Song);; National Forensic Service, Chilgok, South Korea (S. Park);; Seoul National University, Seoul, South Korea (B. Park)

**Keywords:** coronavirus, porcine epidemic diarrhea virus, porcine epidemic diarrhea virus variant, PEDV, large genomic deletion, South Korea, viruses

## Abstract

Since 1992, porcine epidemic diarrhea virus (PEDV) has been one of the most common porcine diarrhea–associated viruses in South Korea. We conducted a large-scale investigation of the incidence of PEDV in pigs with diarrhea in South Korea and consequently identified and characterized a novel PEDV variant with a large genomic deletion.

Porcine epidemic diarrhea virus (PEDV) (family *Coronaviridae*, subfamily *Coronavirinae*, genus *Alphacoronavirus*) is an enveloped, positive-sense, single-stranded RNA virus. PEDV causes an acute and highly contagious enteric disease characterized by severe diarrhea, dehydration, and a high death rate in pigs that results in substantial economic losses in the swine industry (*1*). PEDV was first reported in Belgium and the United Kingdom in 1978; since then, it has been identified in many swine-raising countries in Europe and Asia, notably Belgium, Hungary, Italy, South Korea, Thailand, Japan, and China (*1*,*2*). PEDV was not reported in North and South America until 2013, when it was officially confirmed in the United States; it is spreading rapidly across the country (*3*). We report the emergence and genetic characterization of a novel PEDV variant with a large genomic deletion, which was serendipitously recognized in fecal and intestinal samples of suckling pigs with diarrhea in South Korea as a result of a systematic surveillance program to monitor activity for porcine diarrhea–associated viruses.

## The Study

A total of 2,634 fecal and intestinal samples were collected from pigs exhibiting diarrhea from 569 swine farms in all 9 provinces of South Korea, during January 1–December 31, 2008; age groups of the pigs are defined in the Table. All samples were processed as 10% (vol/vol) suspensions with phosphate-buffered saline (PBS; 0.1 M, pH 7.2), and viral RNA was extracted from them. Subsequently, reverse transcription PCR (RT-PCR) was performed by using 3 primer pairs as described previously (*4*). To determine complete spike (S) gene sequence, we purified, cloned, and sequenced PCR products on an automated DNA sequencer by using T7, SP6 primers, and newly designed S gene–specific primers (primer sequences available on request). Sequences were analyzed by ClustalX version 1.83 program (http://www.clustal.org) and MegAlign software (DNAStar Inc., Madison, WI, USA), and compared with those of reference strains in GenBank. Phylogenetic analysis was conducted with MEGA version 5.22 (*5*). The complete S gene sequence of the PEDV variant with a large genomic deletion (strain MF3809/2008/South Korea) described here has been deposited in GenBank under accession no. KF779469.

**Table:** Incidence of PEDV RNA in diarrhea samples from pigs, South Korea, January 1–December 31, 2008*

Pig age	No. specimens positive/no. tested (%).		
	Feces	Intestine	Total
Suckling pigs, <3 wk	90/526 (17.1)	26/166 (15.7)	116/692 (16.8)
Weaned pigs, >3 wk to 6 wk	12/388 (3.1)	0/116 (0)	12/504 (2.4)
Grower/finisher pigs, >6 wk to 22 wk	29/600 (4.8)	0/208 (0)	29/808 (3.6)
Sows, >1 y	34/140 (24.3)	0/1 (0)	34/141 (24.1)
Boars, >8 mo	1/6 (16.7)	0	1/6 (16.7)
Unknown	13/402 (3.2)	0/81 (0)	13/483 (2.7)
Total	179/ 2062 (8.7)	26/572 (4.5)	205/2634 (7.8)

*All animals showed signs of diarrhea at the time of sample collection. The PEDV variant with a large deletion in the S gene was found in 3 samples (2 fecal and 1 intestinal) of the suckling pigs with diarrhea at 1 farm in Chungnam Province. Other pigs on the same farm were infected with PEDV with the full-length S gene; however, in no instance were both the PEDV variant and PEDV with the full-length S gene observed in 1 pig. PEDV, porcine epidemic diarrhea virus; S gene, spike gene.

Of the 2,634 samples, 205 (7.8%; 49/569 [8.6%] farms) were positive for PEDV: 116 (16.8%) of 692, 12 (2.4%) of 504, 29 (3.6%) of 808, 34 (24.1%) of 141, 1 (16.7%) of 6, and 13 (2.7%) of 483 samples from the 6 age groups tested (Table); however, when SF2/SR2 primers were subjected to PCR, a strong and single band of unexpected size (≈1,000 bp) was found in each PCR product from 3 diarrhea samples of the suckling pigs on 1 farm. Exact length of the band was 981 nt, and the band was much shorter than that of intact fragment because of 612-nt deletion at positions 22777–23388 (1,593 vs 981 nt for PEDV reference strains and PEDV variant, respectively; Technical Appendix Figure 1). Sequence similarity of the 981-nt fragment of MF3809 was found to be in PEDV S gene region in GenBank by BLAST analysis (http://blast.ncbi.nlm.nih.gov/Blast.cgi). The complete S gene (3549-nt segment, corresponding to 1182 aa) of MF3809 had high (93.3%–98.5% nt, 92.0%–98.0% aa) sequence identity to all known PEDV strains for which full-length S gene sequences were available in GenBank, except that MF3809 has the large deletion in its S gene. Phylogenetic analysis confirmed that MF3809 belonged to a cluster containing a PEDV reference strain, not a cluster that included any other coronaviruses, and showed the closest genetic relationship with PEDV strains from South Korea in 2009 (Figure).

**Figure:**
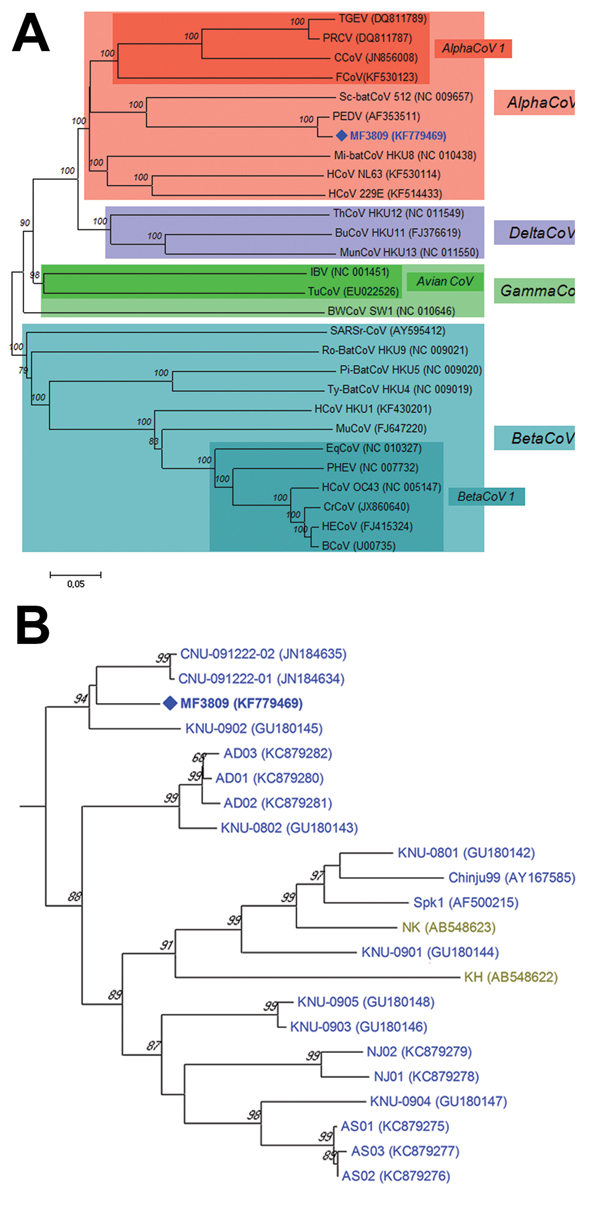
A) Relationships between the PEDV variant (MF3809/2008/South Korea) and other coronaviruses based on the full-length spike gene. PEDV, porcene epidemic diarrhea virus; TGEV, transmissible gastroenteritis virus; PRCV, porcine respiratory coronavirus; CCoV, canine coronavirus; FCoV, feline coronavirus; HCoV, human coronavirus; Mi-batCoV, *Miniopterus* bat coronavirus; Sc-batCoV, *Scotophilus* bat coronavirus; HECoV, human enteric coronavirus; BCoV, bovine coronavirus; PHEV, porcine hemagglutinating encephalomyelitis virus; EqCoV, equine coronavirus; CrCoV, canine respiratory coronavirus; MuCoV, murine coronavirus; Pi-BatCoV, *Pipistrellus* bat coronavirus; Ro-BatCoV, Rousettus bat coronavirus; SARSr-CoV, severe acute respiratory syndrome-related coronavirus; Ty-BatCoV, Tylonycteris bat coronavirus; IBV, infectious bronchitis virus; TuCoV, turkey coronavirus; BWCoV, Beluga whale coronavirus; BuCoV, bulbul coronavirus; ThCoV, thrush coronavirus; MunCoV, munia coronavirus. B) Phylogenetic tree of the entire spike genes of the PEDV variant and all known PEDV strains available in GenBank. The phylogenetic tree was constructed using the neighbor-joining clustering method in MEGA version 5.22 with a pairwise distance (*5*). Bootstrap values (based on 1,000 replicates) for each node are given if >60%. Scale bar indicates nucleotide substitutions per site. PEDV strains isolated from various countries are marked with colors as follows: Europe (black), China (red), Japan (olive green), USA (bright magenta) and South Korea (blue). PEDV, porcine epidemic diarrhea virus.

MF3809 had numerous sequence variations in the S protein (Technical Appendix Table). Besides a large (204-aa) deletion at positions 713–916, a 2-aa (D/NI) deletion was identified at positions 163–164. We also discovered 2 separate insertions: a 4-aa (QGVN) insertion at positions 59–62 and a 1-aa (N) insertion at position 140. A total of 15 separate substitutions were identified, and the number(s) of replaced amino acids ranged from 1 through 5. These sequence variations were similar to those in highly virulent isolates recently reported in China (*6*) and the United States (*3*), as well as in South Korea.

To further characterize the virus, we conducted RT-PCR with respect to the membrane (M) gene using the protocol described previously (*7*). The complete M gene sequence was determined and then submitted, together with the complete S gene sequence, to GenBank under accession no. KF779470. The entire M gene of MF3809 had 96.6%–100% nt (96.0%–100% aa) sequence identity to all known PEDV strains available in GenBank, and phylogenetic analysis showed that MF3809 belonged to a cluster containing PEDV reference strain and showed the closest genetic relationship with 2007 Korean PEDV strains (data not shown).

The filtered samples positive for PEDV variant were inoculated onto Vero cells. After 3 serial passages, no obvious cytopathic effect in Vero cells was noted. Cells and supernatants in every passage were collected separately for RNA extraction and used to detect the virus and determine the amount of the viral RNA in the medium with real-time RT-PCR (*8*). The cells and supernatants of the 3 passages were positive for the virus, and the control inoculated with PBS was negative; however, the amount of viral RNA in the medium decreased with each passage. Whether the positive result was attributed to residual viruses of the initial inoculation or to the decreased propagation of the virus in the cells is not clear. Further studies, such as continuous serial passages and neutralization assays, are needed to determine the final activity of the virus in Vero cells.

## Conclusions

Our large-scale study of the incidence of PEDV in pigs with diarrhea in South Korea found that 7.8% of animals were infected with the virus. Moreover, our investigation identified and characterized a new PEDV variant with a 612-nt deletion in S gene, corresponding to a 204-aa deletion. The coronavirus S protein plays a pivotal role in regulating interactions with specific host cell receptor glycoproteins to mediate viral entry and stimulate induction of neutralizing antibodies in the natural host (*1*,*2*,*9*). Mutations or deletions in the coronavirus S gene affect its pathogenicity and tissue tropism (*10*–*12*). Porcine respiratory coronavirus (PRCV), a naturally occurring deletion mutant of transmissible gastroenteritis virus (TGEV), is an example of pathogenic change and tropism switching, apparently associated with S gene change. PRCV has a 224-aa deletion at positions 21–244 in the N terminal region, which is needed for the enteric tropism of TGEV and comprises antigenic sites C and B (*13*–*15*), of S1 compared with TGEV. In other words, TGEV, a highly enteropathogenic porcine coronavirus, is turned into PRCV, a respiratory pathogen with reduced pathogenicity, as a consequence of a large deletion in the S gene. Unlike PRCV, the PEDV variant has a 204-aa deletion at positions 713–916 in the C-terminus of S1 and N terminus of S2, destroying 4 N-linked glycosylation sites at positions 728, 745, 783, and 875, as well as 2 neutralizing epitopes, SS2 (753–760) and SS6 (769–776) (Technical Appendix Figure 2). These amino acid mutations might cause the conformational change of S protein and result in antigenicity/immunogenicity alteration of the PEDV variant. However, how the PEDV variant was generated and has evolved is not clear. Further studies should be conducted to analyze extensive genomic sequences and determine biological properties, such as pathogenicity, tissue tropism, and transmissibility of the new PEDV variant.

Technical Appendix Summary of amino acid mutations in spike (S) protein of porcine epidemic diarrhea virus (PEDV) variant; schematic diagram of the S genes of classical PEDV, recent prevalent PEDV and the PEDV variant; and alignment of amino acid sequences of the entire S proteins of the PEDV variant and reference strains.
