# Supplementary material for: Novel Porcine Epidemic Diarrhea Virus Variant with Large Genomic Deletion, South Korea
Source: Emerg Infect Dis. 2014 Dec;20(12):2089–92. doi: 10.3201/eid2012.131642 (PMC4257805; doi:10.3201/eid2012.131642)
Supplement: Technical Appendix — Summary of amino acid mutations in spike (S) protein of porcine epidemic diarrhea virus (PEDV) variant; schematic diagram of the S genes of classical PEDV, recent prevalent PEDV and the PEDV variant; and alignment of amino acid sequences of the entire S proteins of the PEDV variant and reference strains. [file 13-1642-Techapp-s1.pdf]

Article DOI: http://dx.doi.org/10.3201/eid2012.131642

Novel Porcine Epidemic Diarrhea Virus Variant with Large Genomic Deletion, South Korea

Technical Appendix

Technical Appendix Table. Summary of amino acid mutations in spike protein of porcine epidemic diarrhea virus variant

Mutations, no. changed amino acids	Amino acid changes	Location in amino acid*
Substitutions		
3	QST → SAN	27–29
3	MNS → GEN	56–58
5	GTGIE → AGQHP	68–72
1	Y → H	84
2	DS → RG	86–87
1	Q → H	89
1	V → A	138
4	R(Q)DGK → SEHS	159–162
1	R → K	197
2	R(K)S → GG	202–203
1	T → E	211
1	T(S) → I	237
1	D → E	247
1	T → S	554
1	D → V	571
Insertions		
4	QGVN	59–62
1	D	140
Deletions		
2	D(N)I	163–164
204	DD(G)I-TVD	713–916

*Classical strain, CV777 (GenBank accession no. AF353511) was used to number the amino acid locations.

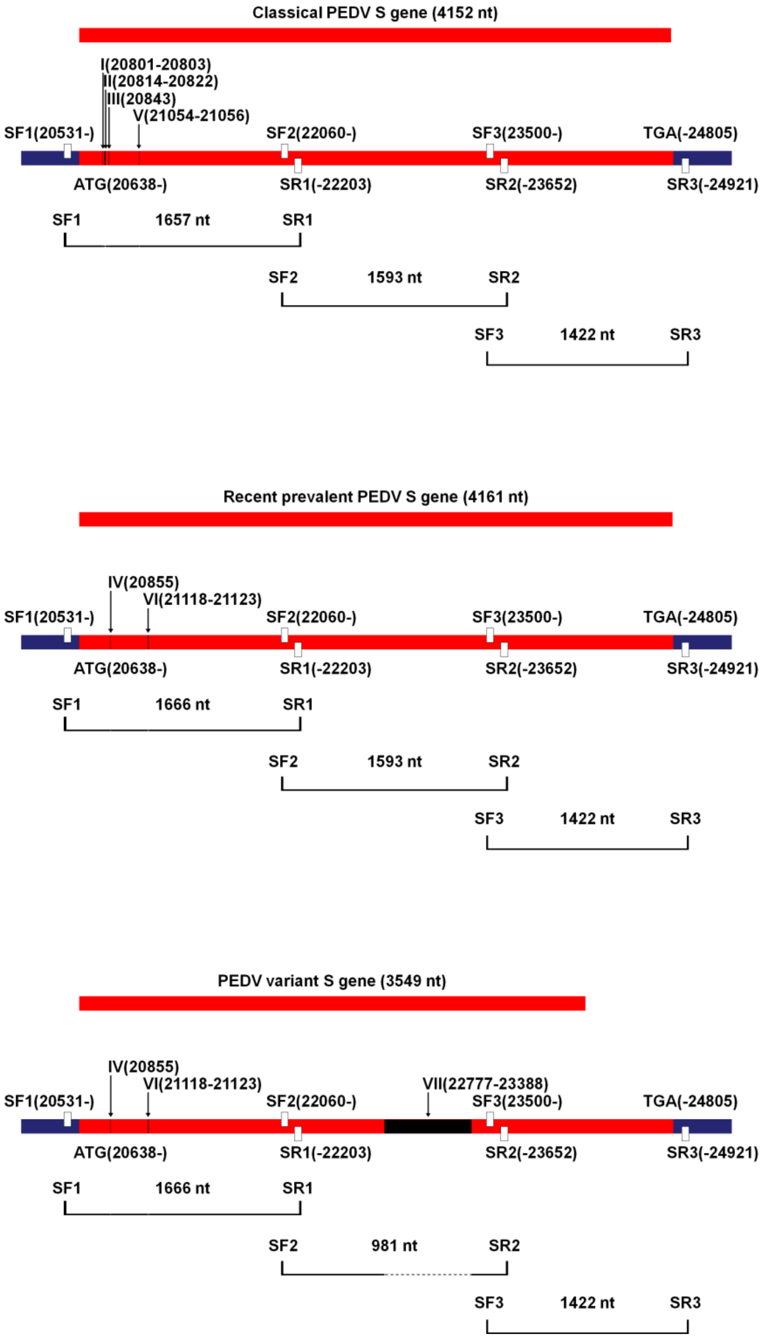

Technical Appendix Figure 1. Schematic diagram of the S genes of classical PEDV, recent prevalent procine epidemic diarrhea virus (PEDV) and PEDV variant. cDNA clones for the entire spike (S) gene of classical PEDV, recent prevalent PEDV and PEDV variant were constructed by reverse transcription PCR using pairs of sense (SF) and antisense (SR) primers: diagrammatic representation of the S gene (red

rectangle) of viral RNA (blue rectangle) show primer binding sites (small open rectangle). Nucleotide deletions (black rectangle) are indicated by arrow and labeled above the arrow as Roman numbers, I–VII: I (20801–20803), II (20814–20822), III (20843), IV (20855), V (21054–21056), VI (21118–21123), VII (22777–23388). Nucleotide numbers shown in diagram correspond to those of the classical strain, CV777 (GenBank accession no. AF353511).

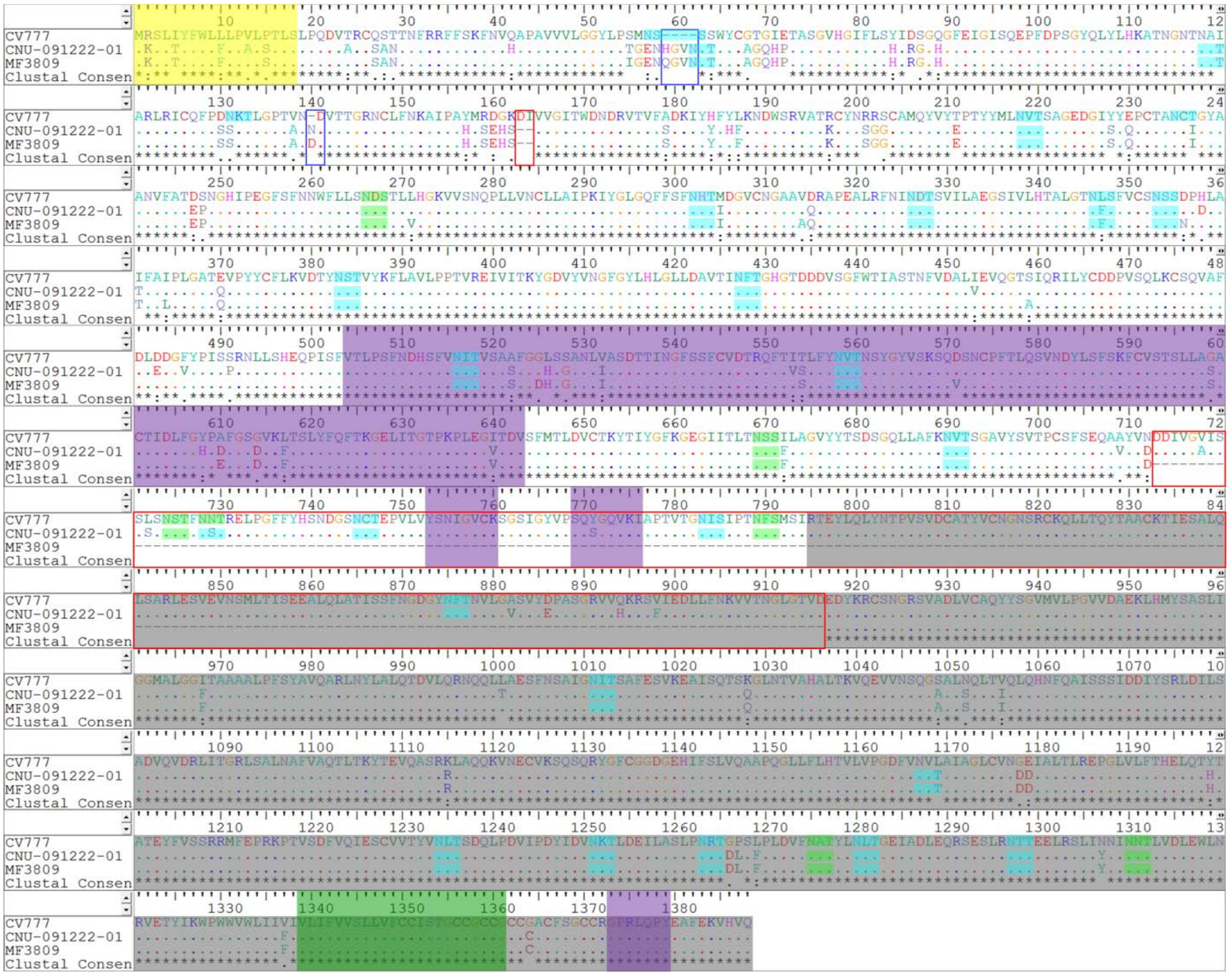

Technical Appendix Figure 2. Alignment of amino acid sequences of the entire spike (S) proteins of porcine epidemic diarrhea virus (PEDV) variant and reference strains. Classical strain, CV777 (GenBank accession no. AF353511), was used as the reference for numbering. CNU-091222–01 (GenBank accession no. JN184634), the closest relative strain of the PEDV variant, was used for better

understanding. Dots represent amino acids that are identical to those of the CV777. Yellow, purple, green, and gray shadows indicate the locations of signal peptide (1–18 aa), 4 neutralizing epitopes (COE, 504–643 aa; SS2, 753–760 aa; SS6, 769–776 aa; 2C10, 1373–1379 aa), transmembrane anchor (1339–1361 aa), and S2 domain (795–1388 aa). Blue and red rectangles stand for the inserted and deleted amino acids compared with the CV777. Both yellow green and mint green shadows represent the Asn-Xaa-Ser/Thr sequons, but only mint green represent asparagines predicted to be N-glycosylated.
